# Pyroptosis burden is associated with anti-TNF treatment outcome in inflammatory bowel disease: new insights from bioinformatics analysis

**DOI:** 10.1038/s41598-023-43091-0

**Published:** 2023-09-22

**Authors:** Xin Gao, Chen Wang, Xiao-Tong Shen, Chen-Yang Li, Yan-Chen Li, He Gao, Jia-Ming Qian, Xiao-Lan Zhang

**Affiliations:** 1 Department of GastroenterologyHebei Key Laboratory of GastroenterologyHebei Clinical Research Center for Digestive Diseases, The Second Hospital of Hebei Medical University, Hebei Institute of Gastroenterology, Shijiazhuang, 050035 Hebei China; 2 Department of Gastroenterology, Peking Union Medical College Hospital, Chinese Academy of Medical Sciences, Beijing, 100730 China

**Keywords:** Computational biology and bioinformatics, Immunology, Biomarkers

## Abstract

Biological agents known as anti-tumor necrosis factor (TNF) drugs are frequently utilized in the treatment of inflammatory bowel disease (IBD). In this study, we analyzed the shared processes of pyroptosis in Ulcerative colitis (UC) and Crohn's disease (CD), as well as explored the correlation between the burden of pyroptosis and the results of anti-TNF treatment based on bioinformatics analyses. We identified *CAPS1*, *CASP5*, *GSDMD*, *AIM2*, and *NLRP3* as the hub genes, with AIM2 being the most effective indicator for predicting the response to anti-TNF therapy. We also noticed that non-responders received anti-TNF therapy exhibited elevated AIM2 protein expression. Subsequently, we conducted a cluster analysis based on AIM2-inflammasome-related genes and discovered that patients with a higher burden of AIM2 inflammasome displayed stronger immune function and a poor response to anti-TNF therapy. Overall, our study elucidates the pathway of pyroptosis in IBD and reveals AIM2 expression level as a potential biomarker for predicting the effectiveness of anti-TNF therapy.

## Introduction

Inflammatory bowel disease (IBD), consisting of ulcerative colitis (UC) and Crohn's disease (CD), affects 6 to 8 million people worldwide. IBD, as a chronic, progressive, and recurring intestinal disorder, has a significant impact on patients' quality of life and daily activities. Although it is widely accepted that IBD is caused by the abnormal immune response to microbiota in genetically predisposed individuals^1^, the exact pathogenesis is still unknown.

Recent advances in the understanding of the mechanisms underlying the pathogenesis of IBD have enabled the development of treatments targeting the fundamental immune processes. Anti-TNF drugs are the most classic biological agent for the treatment of IBD, but up to 30% of patients are unresponsive to initial treatment and up to 50% lose response over time^2–4^. This means that in addition to the TNF pathway, there are other important inflammatory pathways involved in the occurrence of IBD. Consequently, a comprehensive and deeper understanding of the functioning of the intestinal immune system and the immunopathogenesis of IBD could help us to develop immunotherapeutic agents for IBD, providing new avenues for immune treatments of this condition.

Gasdermin-mediated pyroptosis is a novel cell death form that is accompanied by the release of a large amount of inflammatory mediators and has been recognized as the major regulator of host inflammation^5^. Gasdermin proteins are cleaved in response to different upstream signals, with the activation of inflammasomes leading to the cleavage of Gasdermin D (GSDMD) and the release of IL-1β and IL-18 being one of the most extensively studied pathways^6,7^. Specifically, The typical inflammasome is a multimeric complex usually composed of sensor proteins, adapter proteins, and effector proteins. Sensors can be classified into two main protein families: the NOD-like receptor (NLR) protein family, which includes NLRP1, NLRP2, NLRP3, NLRP6, NLRP7, NLRC4, and potentially NLRP12; and the pyrin and HIN domain-containing (PYHIN) protein family, such as absent in melanoma (AIM2). Pattern recognition receptors (PRRs) recognize and detect pathogen-associated molecular patterns (PAMPs) and damage-associated molecular patterns (DAMPs) to initiate the inflammasome signaling pathway. The activated receptor protein oligomerizes and binds to the adapter protein, and then recruits the effector protein procysteinyl aspartate specific protease-1 (procaspase-1) to form a typical inflammasome complex. Subsequently, procaspase-1 is activated and forms a dimer to become a mature cleaved caspase-1, inducing a series of inflammatory responses. Activated caspase-1 cleaves GSDMD to generate GSDMD-N, which subsequently forms a pore in the cell membrane, leading to cell swelling and pyroptosis. This pathway has been implicated in the development of a number of inflammatory diseases, including gout, atherosclerosis, and Alzheimer's disease^8–11^. As the TNF pathway is linked to pyroptosis, it is plausible to hypothesize that the level of pyroptosis may have an impact on the effectiveness of anti-TNF therapy.

In this study, we analyzed co-altered pyroptosis-related genes in patients with ulcerative colitis and Crohn's disease to profile pyroptosis patterns. Based on our research, anti-TNF therapy has shown some potential in reducing pyroptosis. However, it may not be effective for patients with a high pyroptosis burden. Our proposal suggests that the expression level of AIM2 could be a useful factor in categorizing IBD patients, and that patients with a high expression of AIM2 may not be suitable candidates for anti-TNF treatment.

## Materials and methods

### Datasets collection

**Figure 1:**
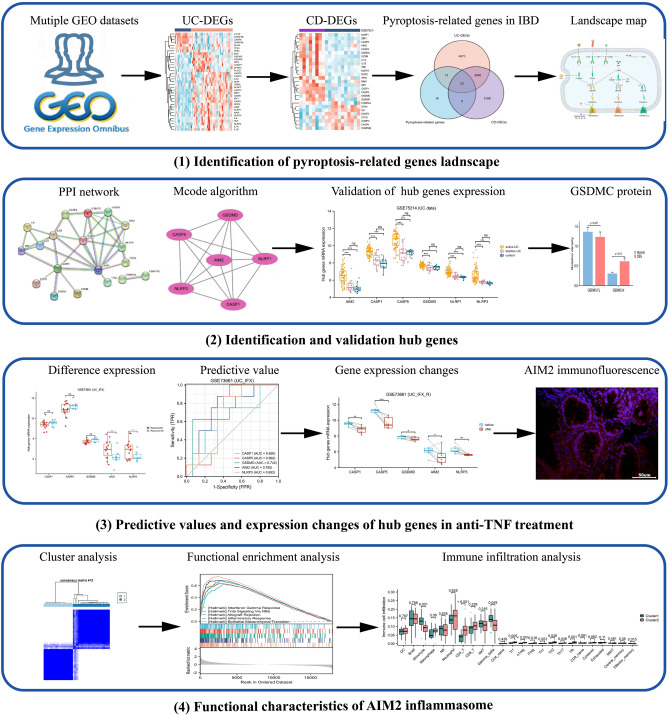
Research flow chart of this study. *GEO* gene expression omnibus, *UC* ulcerative colitis, *CD* Crohn’s disease, *DEGs* differentially expressed genes, *IBD* inflammatory bowel disease, *PPI* protein–protein interaction network.

The flowchart of this study is shown in Fig. 1. The microarray data and corresponding clinical information of IBD patients were obtained from seven Gene Expression Omnibus datasets (GEO, https://www.ncbi.nlm.nih.gov/gds)^12–17^. All databases are depicted in Table 1. In brief, GSE47908, GSE38713, and GSE107499 were utilized to analyze the mRNA expression differences between IBD patients and controls, meanwhile GSE12251, GSE73661, GSE16879, and GSE98820 were used to investigate the correlation between hub genes expression and anti-TNF therapy outcomes.

**Table 1:** The information of all GEO cohorts.

Dataset	Platform	Sample for this study/Disease	Anti-TNF therapy	Details
GSE47908 (12)	GPL570	39 Ulcerative colitis	No	–
		15 Healthy controls		
GSE75214 (13)	GPL6244	97 Ulcerative colitis	No	–
		8 Crohn’s colitis		
		11 Healthy controls		
GSE20881 (14)	GPL1708	82 Crohn’s colitis	No	–
		67 Healthy controls		
GSE12251 (15)	GPL570	22 Ulcerative colitis	Yes	R: 12; NR: 11 (one NR provided two specimens)
GSE73661 (16)	GPL6244	23 Ulcerative colitis	Yes	R: 8; NR: 15
GSE16879 (17)	GPL570	24 Ulcerative colitis	Yes	UC (R: 8; NR: 16)
		19 Crohn’s colitis		CD (R: 12; NR: 7)
GSE98820	GPL17692	10 Crohn's disease	Yes	42 inflammatory tissues from 10 CD patients (all patients were responders to adalimumab)

### Identification of pyroptosis-related differentially expressed genes

For GSE47908, the differentially expressed genes (DEGs) between inflammatory tissues and healthy tissues were found using the “limma” package^18^. GSE75214, the DEGs between inflammatory tissues and healthy tissues were identified using GEO2R (https://www.ncbi.nlm.nih.gov/geo/geo2r/). The adjusted *p*-value < 0.05 was statistically significant. 58 genes involved in pyroptosis are shown in Table S1. Then we intersected the DEGs from two databases with 58 pyroptosis-related genes by Venn diagram tool.

### Protein–protein interaction network construction and evaluation of hub genes

We investigated interactions between DEGs using a protein–protein interaction network (PPI network, https://string-db.org), obscuring individual target protein nodes and defining statistical significance as an interaction score > 0.9. Then we used the molecular complex detection (MCODE) plug-in in Cytoscape software to identify the hub genes. Hub genes were considered as the modules having network scores larger than 5.

### Validation of hub genes expression

The identified hub genes' expression levels were verified in GSE75214 for UC and GSE20881 for Crohn’s colitis (CDc). The comparison of the two datasets was performed through the Wilcoxon rank sum test.

### Predictive value of hub genes for anti-TNF therapy

We used receiver operating characteristic curves (ROC) and the area under the curve (AUC) by the “pROC” package to estimate the diagnostic and efficacy predictive value of pyroptosis-related genes.

### Classification of IBD patients based on AIM2 inflammasome-related genes

AIM2 inflammasome-related genes were obtained from the GSEA database (AIM2_INFLAMMASOME_COMPLEX), including *AIM2*, *CAPS1*, *CASP12*, *CASP4*, *PYCARD*, as described before^19^. Yet, most individuals have a frameshift mutation in the CASP12, resulting in premature transcriptional termination and translation of an inactive truncated protein^20^, moreover, research has shown that CASP12 does not act as an inflammasome negative regulator^21^. Therefore, We identified *AIM2*, *CAPS1*, *CASP4*, and *PYCARD* as AIM2 inflammasome-related genes. Then, the “ConsensusClusterPlus” package was used for cluster analysis^22^ and DEGs between the different clusters were identified by the “limma” package.

### Functional enrichment analysis

Based on the DEGs between different clusters, Gene ontology (GO), Kyoto Encyclopedia of Genes and Genomes (KEGG, www.kegg.jp/kegg/kegg1.html), and Gene Set Enrichment Analysis (GSEA) were performed using the “clusterProfiler” package^23^, and the “Hallmarks” pathway was selected for GSEA.

### Immune landscape analysis

We used ImmuCellAI (http://bioinfo.life.hust.edu.cn/ImmuCellAI#!/)^24^ to evaluate the difference in immune cell infiltration between different clusters. “GSVA” package^25^ was used to assess the alternations of immune-related pathways.

### Construction of miRNA-mRNA regulatory network

The miRNA-mRNA regulatory network was downloaded from the miRNet database (https://www.mirnet.ca/miRNet/home.xhtml)^26^ and visualized using Cytoscape software.

### Western blot

This study was approved by the Research Ethics Committee of the Second Hospital of Hebei Medical University (ethics approval NO. 2023-AE079). All animal experiments in this study were conducted in accordance with ARRIVE guidelines for animal reporting. All methods are carried out in accordance with relevant guidelines and regulations. Male C57BL/6 mice aged 6–8 weeks (weighing 18–22 g) were purchased from Beijing Vital River Laboratory Animal Technology Co. Ltd. and housed under specific pathogen-free conditions. Acute colitis was induced by oral administration of 2% Dextran Sulphate Sodium (DSS) in drinking water for 7 days, and control mice received water without DSS (normal group, n = 4). On day 8, the mice were sacrificed and colon samples were collected. Protein was extracted from the colon tissue using RIPA lysis buffer (Solarbio, China). Protein concentrations were measured using the BCA Protein Assay Kit (Solarbio, China). Equal amounts of protein (25 µg per lane) were separated by 10% SDS-PAGE and transferred onto polyvinylidene fluoride (PVDF) membranes (Millipore Corp, USA). The PVDF membranes were then blocked with 5% milk for 1 h and incubated with primary antibodies against GSDMC (Abclonal, Cat# A16741, RRID: AB_2769696, China^27^) overnight at 4 °C. The protein bands were detected using the Odyssey CLx imaging systems (Li-COR Biosciences, Lincoln, NE, USA), and the band strength was quantified by image-J software.

### Patients and immunofluorescence

We recruited 20 individuals with ulcerative colitis and obtained paraffin-embedded tissue samples from endoscopic biopsies for further processing into 5-µm-thick frozen sections. All the individuals were admitted to The Second Hospital of Hebei Medical University and were treated with infliximab. This project was reviewed and approved by the Ethics Committee of the Second Hospital of Hebei Medical University (ethics approval NO. 2023-AE080), and the patients provided their written informed consent. All methods are implemented in accordance with relevant guidelines and regulations. Following dewaxing with xylene and rehydration with gradient ethanol, tissue slides were washed with deionized water. Antigenic repair was performed using microwaves and repair solutions. Tissues were blocked with 10% normal goat serum for 1 h prior to incubation with AIM2 antibody antibody (Signalway, USA) overnight at 4 °C. We used 4',6-diamidino2-phenyindole (DAPI) to stain the nucleus of samples and subsequently captured images using a fluorescence microscope (Nikon Eclipse C1, Japan). Image-Pro Plus software was used to calculate integral optical density (IOD) and the area ratio (AR) of the positively stained area as semi-quantitative values of the expression of AIM2. For each patient, we randomly selected two sites for evaluation, with the standard being that each site contains sufficient epithelial tissue and immune cells.

### Statistical analysis

All statistical analyzes were performed in R software (version 3.6.0). All of the statistical tests performed in the study were two-sided. Wilcoxon rank sum tests were used for data that were not necessarily normally distributed, whereas Student’s t-tests were used for data that were normally distributed. *P* < 0.05 was considered statistically significant.

### Ethics statement

The studies involving animal experiments and human participants were reviewed and approved by Ethics Committee of the Second Hospital of Hebei Medical University. The patients provided their written informed consent to participate in this study.

## Results

### Identification of pyroptosis landscape in patients with inflammatory bowel disease

**Figure 2:**
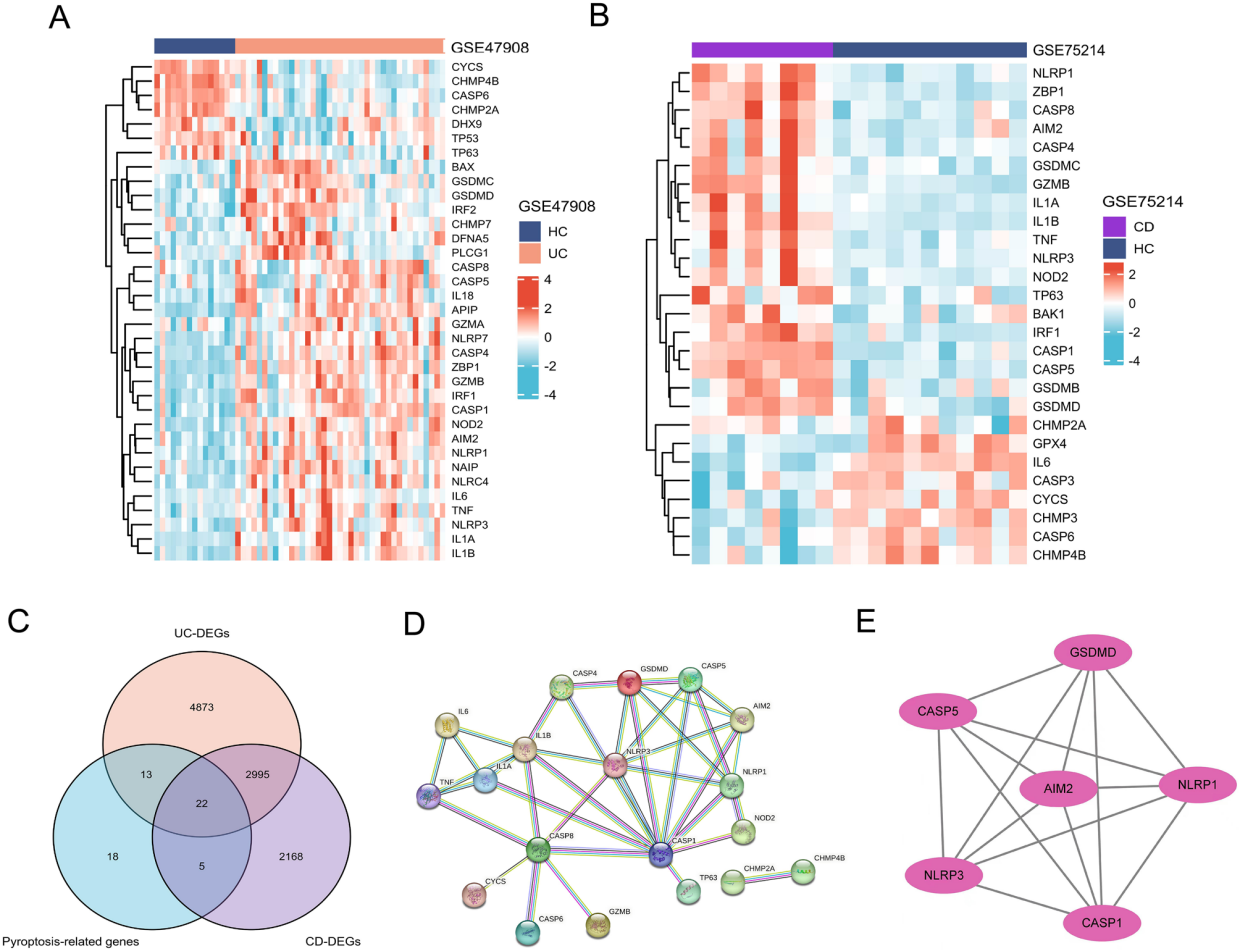
Identification of pyroptosis-related hub genes in IBD. (**A**) and (**B**) The heatmaps illustrate the diferential gene expressions in UC (**A**) and CD (**B**), HC refers to the healthy control group. (**C**) Venn diagrams. (**D**) PPI network. (**E**) Cytoscape plug-in Mcode.

A comparative analyses of mucosal gene expression between individuals with IBD and controls revealed differential expression of 35 pyroptosis-related genes in patients with UC. Among these genes, 7 were found to be downregulated and 28 upregulated (Fig. 2A). Similarly, in patients with CD, 27 pyroptosis-related genes were differentially expressed, with 8 being downregulated and 19 upregulated (Fig. 2B). A total of 22 genes were selected for further investigation based on their overlapping presence in both conditions (Fig. 2C). The mRNA expression of *AIM2, CASP1, CASP4, CASP5, CASP8, GSDMC, GSDMD, GZMB, IL1A, IL1B, IRF1, NLRP1, NLRP3, NOD2, TNF,* and *ZBP1* were increased in both disease state. Then, we used the PPI network to construct interactive relationships of 22 shared DEGs between UC and CD (Fig. 2D). Finally, by using the MCODE plug-in in Cytoscape, we discovered a module with a score of six, consisting of 6 nodes and 15 edges. *AIM2*, *GSDMD*, *CASP5*, *CASP1*, *NLRP3,* and *NLRP1*, upregulating in inflammation, were identified as hub genes (Fig. 2E).

### Elevated expression of GSDMC-N in experimental colitis mice

Gasdermin proteins have been identified as key executioners of pyroptosis, with most Gasdermin-N proteins possessing the ability to form membrane pores. Interestingly, *GSDMC* mRNA expression is elevated in IBD patients (Fig. 2A,B), suggesting that GSDMC-mediated pyroptosis may also be associated with the disease. Therefore, we investigated the expression of the GSDMC protein in experimental colitis mice induced by DSS. The results showed that while the full-length expression of GSDMC did not increase, more GSDMC was cleaved under inflammatory conditions (Fig. S1). The findings indicate that GSDMC could potentially play a role in the development of experimental colitis, and may be a direction for future research.

### Validation of hub genes expression

Next, to verify the expression of hub genes, additional cohorts (GSE75214 for UC and GSE20881 for CD) were utilized. Our findings indicate that the expression of hub genes in quiescent ulcerative colitis patients and healthy controls is similar, but increases in active patients. This suggests that these hub genes could potentially serve as biomarkers for disease activity (Fig. S2). *CASP1, CASP5, AIM2, NLRP3,* and *GSDMD* show good reproducibility and were therefore used for further analysis.

### Expression changes of hub genes during anti-TNF therapy

**Figure 3:**
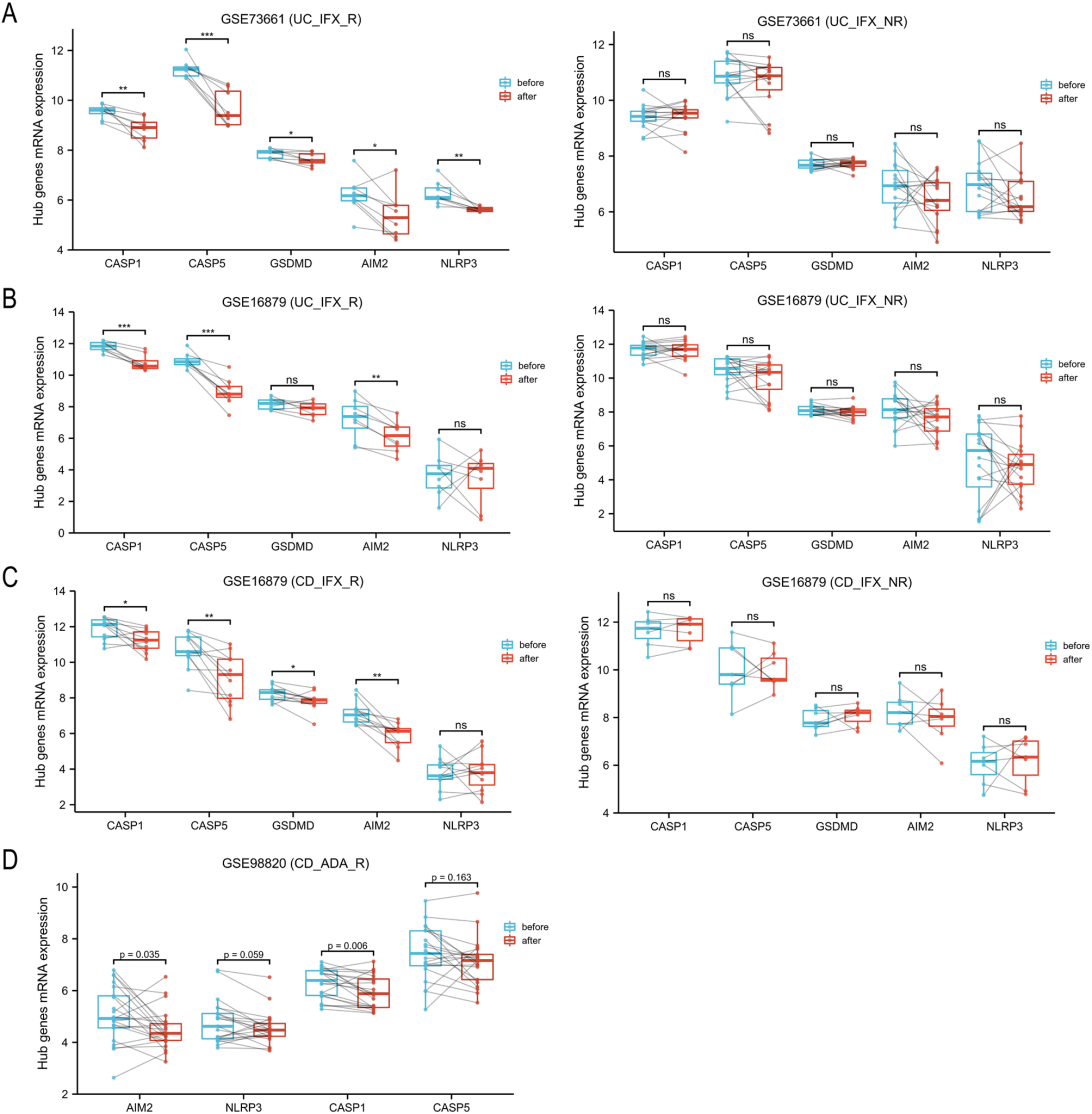
Expression changes of hub genes in inflammatory tissues that underwent anti-TNF therapy. (**A**–**C**) Expression changes of hub genes associated with infliximab therapy in responders or non-responders based on GSE73661 (**A**) and GSE16879 (**B**,**C**). (**D**) Based on the GSE98820, hub genes expression (this dataset do not has *GSDMD* gene) of 42 inflammatory tissues (21 before treatment, 21 after treatment) from 10 CD patients (all patients were responders to adalimumab) are analyzed by paired sample T test. *UC* ulcerative colitis, *CD* Crohn’s disease, *IFX* infliximab, *ADA* adalimumab, *R* responder, *NR* non-responders. *P < 0.05, **P < 0 0.01, ***P < 0.001. *ns* not significant.

The data before and after the initial infliximab treatment were provided by GSE73661 and GSE16879 (Fig. 3A–C). The GSE98820 dataset provided data on adalimumab treatment, and all patients experienced positive effects from the therapy (Fig. 3D). Among non-responders, the expression of hub genes did not change significantly during anti-TNF therapy. However, in most cases, anti-TNF therapy led to a reduction in hub gene expressions in responders. These findings suggest that anti-TNF therapy has the potential to decrease pyroptosis levels in patients with IBD.

### Predictive values of hub genes in anti-TNF treatment

**Figure 4:**
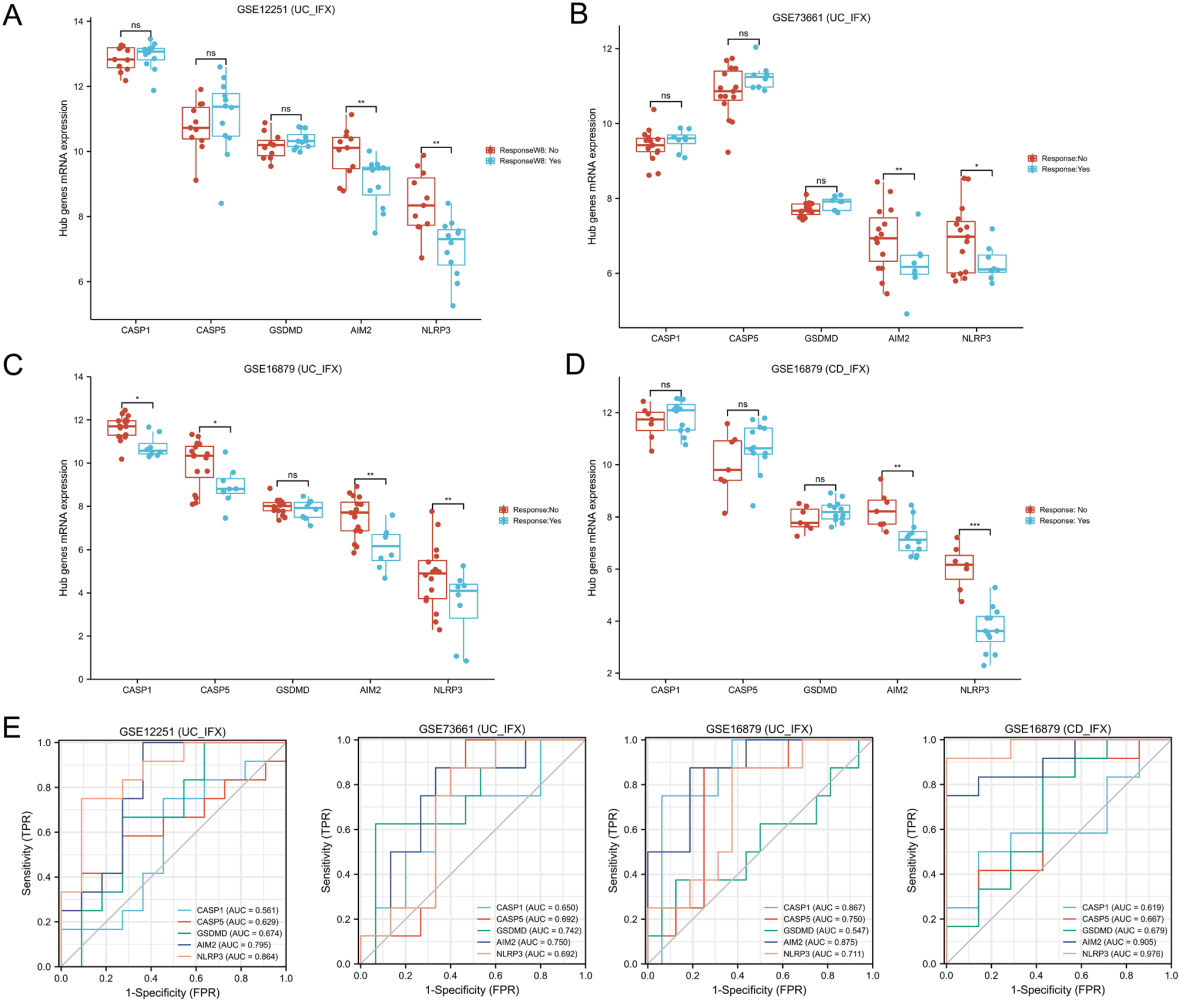
Expression differences of hub genes between responders and non-responders with anti-TNF treatment. (**A**) Based on the GSE12251 dataset, 22 UC patients underwent colonoscopy with biopsy before infliximab treatment. Response to infliximab was defined as endoscopic and histologic healing at week 8. The expression of hub genes was compared according to treatment effect. (**B**) Based on the GSE73661 dataset, 23 UC patients were treated with infliximab for 4–6 weeks. Response was defined as endoscopic mucosal healing. The expression of hub genes is compared according to treatment effect. (**C**) and (**D**) Based on the GSE16879 dataset, 24 UC (**C**) and 18 CDc (**D**) patients were treated with infliximab for 4–6 weeks. According to treatment effect, the expression of hub genes is compared between responders and non-responders. (**E**) ROC curve show the predictive values of hub genes in the anti-TNF treatment. *UC* ulcerative colitis, *CD* Crohn’s disease, *IFX* infliximab. *P < 0.05, **P < 0 0.01, ***P < 0.001. *ns* not significant.

Four datasets (GSE12251, GSE73661, GSE16879, and GSE98820) provided the data for patients who had received the anti-TNF therapy. Specifically, we analyzed the hub genes expression of responders and non-responders at the onset of anti-TNF therapy. Our study revealed that non-responders exhibited higher levels of *AIM2* and *NLRP3* expression compared to responders (Fig. 4A–D). Additionally, the AUCs of AIM2 in all queues were greater than 0.700 (ranging from 0.750 to 0.905, Fig. 4E). These results suggest that a high level of pyroptosis-induced inflammation is associated with unfavorable outcomes for anti-TNF medication and that AIM2 may serve as a reliable predictive biomarker for this clinical therapy choice.

### Protein expression of AIM2 in UC patients receiving anti-TNF therapy

**Figure 5:**
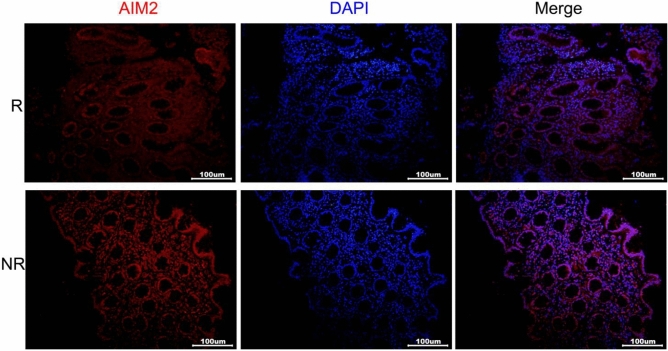
Immunofluorescence analysis of AIM2 protein in UC patients receiving anti-TNF therpay.

We studied the protein expression of AIM2 in 20 UC patients who received infliximab treatment at the Second Hospital of Hebei Medical University, and the demographic data was listed in Table 2. The results of the immunofluorescence analysis showed that AIM2 was expressed in both epithelial cells and interstitial immune cells, as seen in Fig. 5. Notably, non-responders had higher levels of AIM2 protein expression compared to responders. However, there was no observed correlation between AIM2 protein expression and clinical parameters, such as inflammatory markers and disease activity (Figure S3).

**Table 2:** Demographic data of UC patients.

Characteristics	Responders	Non-responders	*P* value
Number (n)	14	6	-
Sex (n, M/F)	11/3	2/4	0.122
Age (years, mean ± sd)	35.9 ± 13.1	35.2 ± 15.0	0.910
Smoking (n)	2	0	1.000
Age at diagnosis (years, mean ± sd)	31.8 ± 10.2	31.7 ± 10.7	0.981
Weight [kg, median (IQR)]	60 (53.75, 74.5)	59 (45.25, 61.5)	0.283
BMI ( mean ± sd)	21.3 ± 3.9	19.3 ± 2.9	0.266
Duration [years, median (IQR)]	2 (0.7, 6.5)	0.7 (0.4, 7.7)	0.385
Hemoglobin (mean ± sd)	110.6 ± 36.3	98.3 ± 23.3	0.457
ESR [median (IQR)]	26.0 (6.5, 34)	13 (6.5, 19.5)	0.710
Albumin (mean ± sd)	34.6 ± 8.1	30.2 ± 8.8	0.291
CRP [median (IQR)]	18.7 (1.3, 57.1)	13.7 (1.8, 24.1)	0.773
PLT [median (IQR)]	239 (204.8, 377.2)	383.5 (229, 536.5)	0.179
UCEIS [median (IQR)]	7 (5, 8)	5.5 (5, 7.5)	0.932
AIM2-IOD/Area	16.3 (15.6, 21.2)	19.8 (17.8, 22.1)	0.033*

### Molecular subtyping based on AIM2 inflammasome-related genes

**Figure 6:**
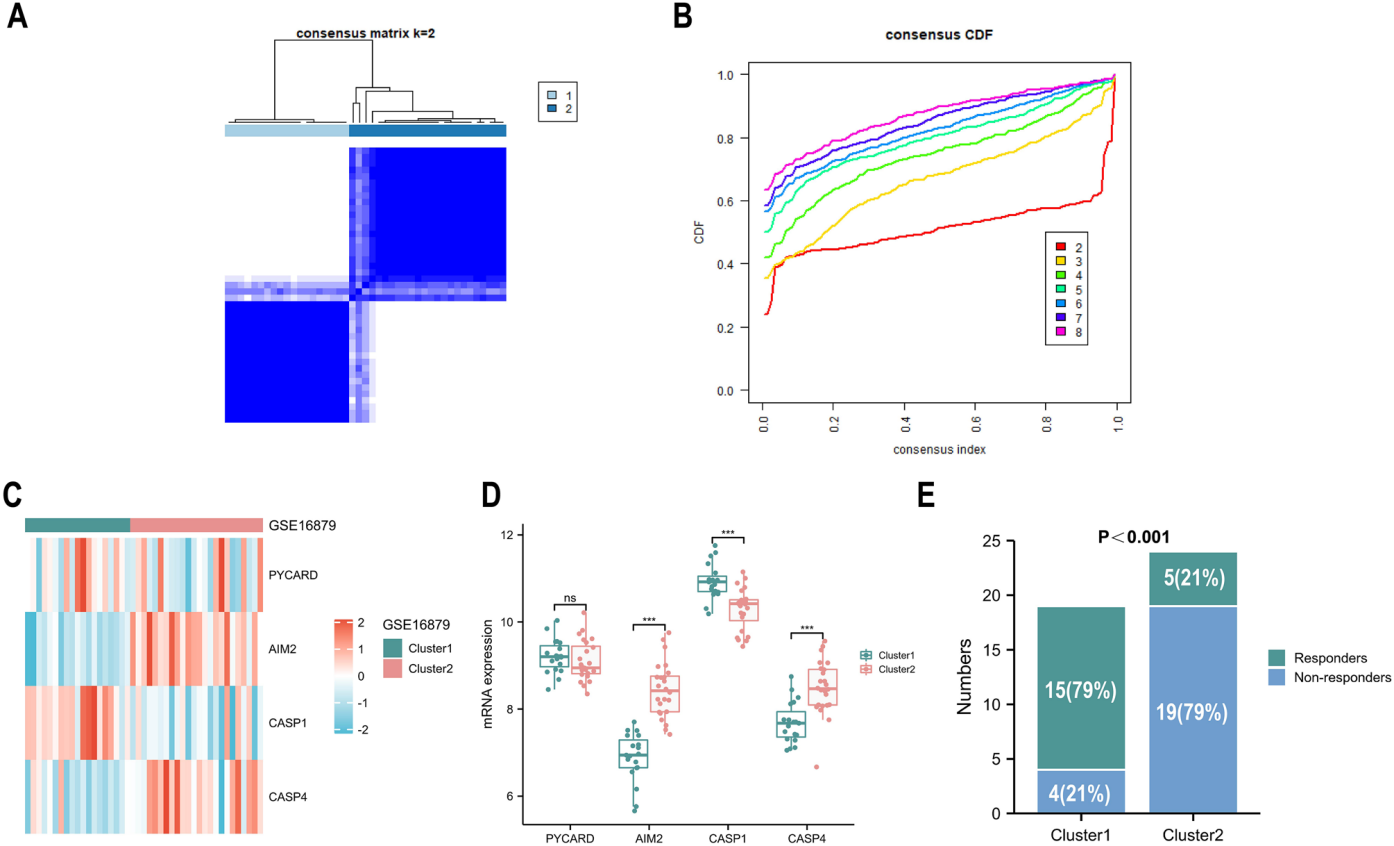
AIM2 inflammasome-related gene-based classification of IBD patients. (**A**) IBD patients of GSE16879 are classified into two clusters based on AIM2 inflammasome-related genes using unsupervised consensus clustering. (**B**) Item-consensus plot demonstrates K = 2 is the optimal clustering. (**C**) and (**D**) Heatmap (**C**) and box plots (**D**) show expressions of AIM2 inflammasome-related genes in each cluster. (**E**) The stacked column chart displays the proportion of responders and non responders in two cluster. *P < 0.05, **P < 0 0.01, ***P < 0.001. *ns* not significant.

We conducted a cluster analysis of patients with inflammatory bowel disease receiving anti-TNF treatment from GSE16879 dataset based on four AIM2 inflammasome-related genes, and the optimal clusters were produced when K = 2 (Fig. 6A,B). *AIM2* and *CASP4* were generally upregulated in cluster 2, meanwhile, *CASP1* was generally downregulated in cluster 1 (Fig. 6C,D). Patients in cluster 1 demonstrate a higher response rate to anti-TNF therapy than patients in cluster 2 (Fig. 6E).

### Functional enrichment analysis

**Figure 7:**
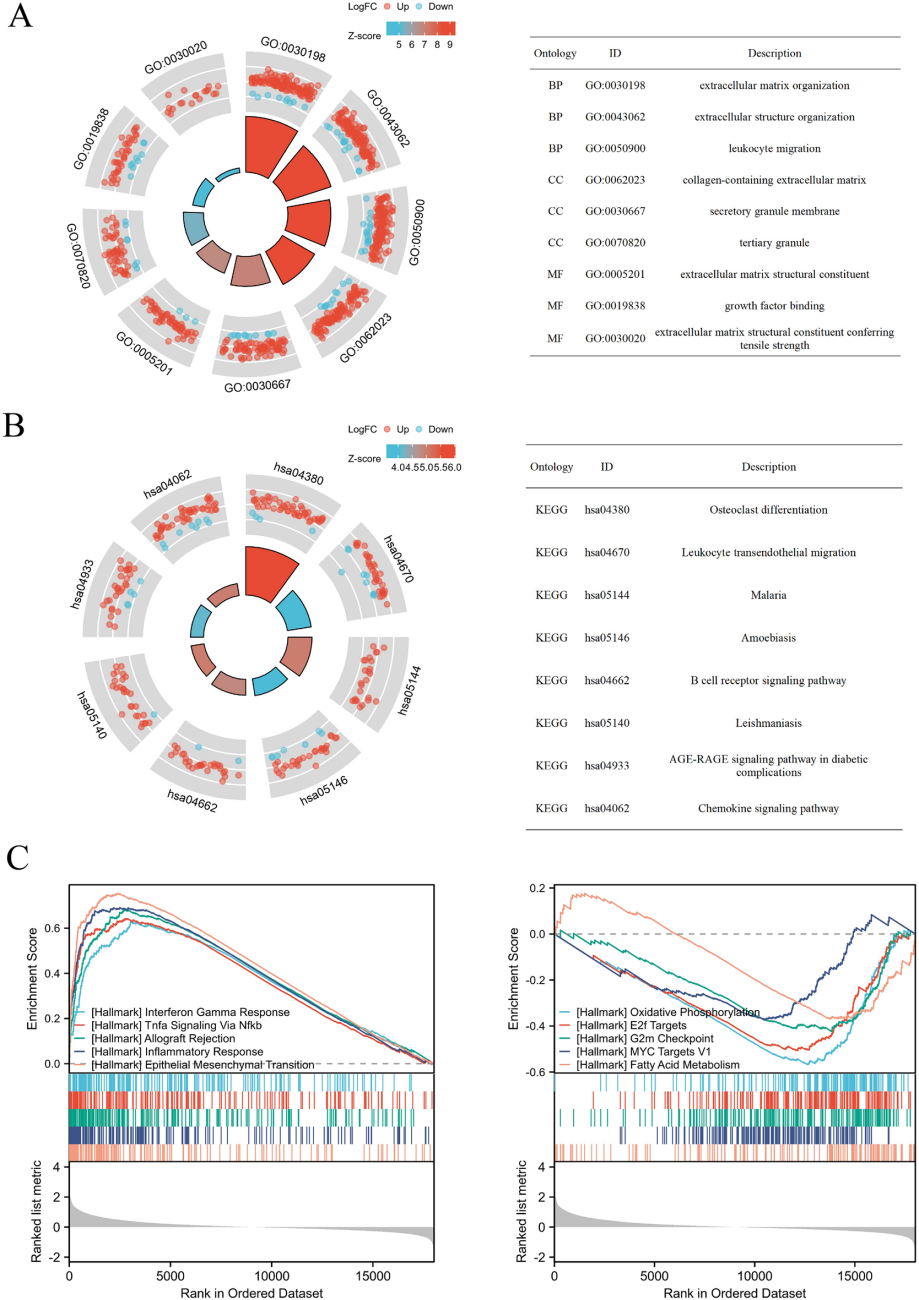
Pathway enrichment analysis. (**A**) The top 3 enriched pathways according to GO analysis (*BP* biological process, *MF* molecular function, *CC* cellular components) (**B**) KEGG pathway analysis (www.kegg.jp/kegg/kegg1.html). (**C**) Enriched pathways according to GSEA analysis.

Further functional enrichment analysis was carried out to elucidate the function differences between the two clusters (Table S2). GO analysis revealed that multiple immune-related pathways, including leukocyte migration, macrophage activation, and CD4-positive, alpha–beta T cell activation were elevated in cluster 2 (Fig. 7A). Leukocyte transendothelial migration and B cell receptor signaling pathway were both active in cluster 2, according to KEGG pathway analysis (Fig. 7B). GSEA results showed INTERFERON_GAMMA_RESPONSE, TNFA_SIGNALING_VIA_NFKB, and INFLAMMATORY_RESPONSE pathway were upregulated in cluster 2, and OXIDATIVE_PHOSPHORYLATION and FATTY_ACID_METABOLISM were upregulated in cluster 1 (Fig. 7C).

### Identification of distinct immunological landscapes between different clusters

**Figure 8:**
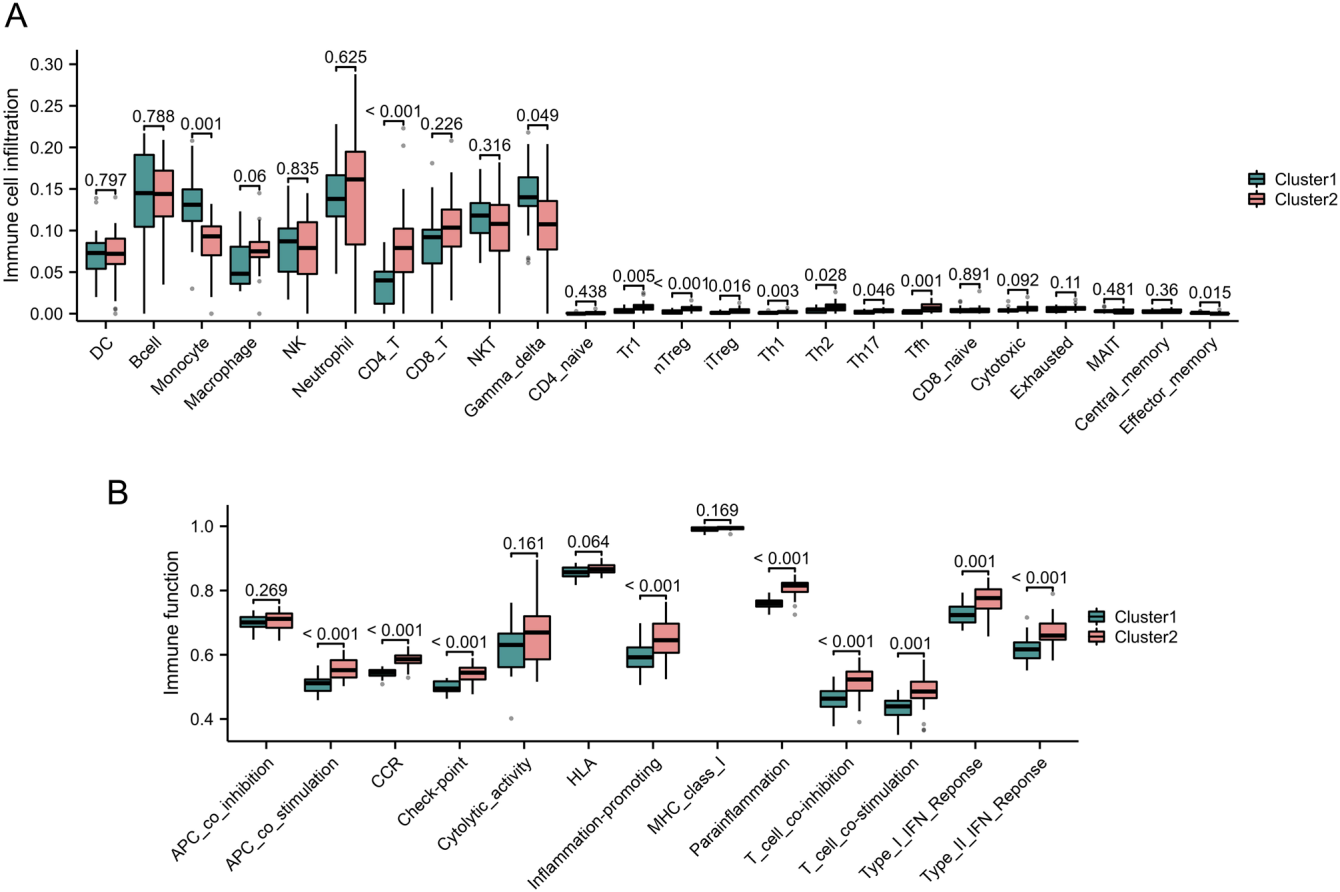
Comparison of immune microenvironment between two clusters. (**A**) and (**B**) Box plots demonstrate immune cell infiltration levels (**A**) and immune reaction (**B**) in two clusters.

The abundance of 24 immune cells was estimated by ImmuCellAI. In general, CD4^+^T cells, TH1, TH2, TH17 and macrophage were elevated in cluster2 (Fig. 8A). Similarly, various immune-related pathways are enriched in cluster 2, such as inflammation-promoting and IFN response (Fig. 8B). In summary, these findings indicated that patients with high AIM2 inflammasome burden have a stronger inflammatory response, which may be one of the reasons for anti-TNF treatment failure.

### Construction of the miRNA-mRNA network

The target microRNAs (miRNAs) of the AIM2 inflammasome-related genes (*AIM2*, *CASP1*, *CASP4*, and *PYCARD*) were predicted using the miRNet program. Ultimately, we collected 119 miRNAs from four inflammasome-related genes (Table S3), with 11, 31, and 29 miRNAs regulating *AIM2*, *CASP1*, and *CASP4*, respectively, and 48 miRNAs regulating *PYCARD*. The resulting miRNA-gene network was visualized using Cytoscape (Fig. S4).

## Discussion

Pyroptosis has been identified as the major regulator of inflammation, with its role in inflammatory bowel disease garnering attention. Such as knockout of GSDMD^8,28^ and GSDME^29^ significantly reduced experimental colitis induced by DSS and 2,4,6-trinitrobenzene sulfonic acid (TNBS), respectively. In actuality, both UC and CD are characterized by inappropriate inflammatory and immune responses, which are thought to be the primary cause driving their pathogenesis and are intimately linked to pyroptosis. Therefore, this study aims to comprehensively elucidate the shared pyroptosis landscape of UC and CD, thus providing a new perspective for clinical diagnosis and treatment of IBD.

**Figure 9:**
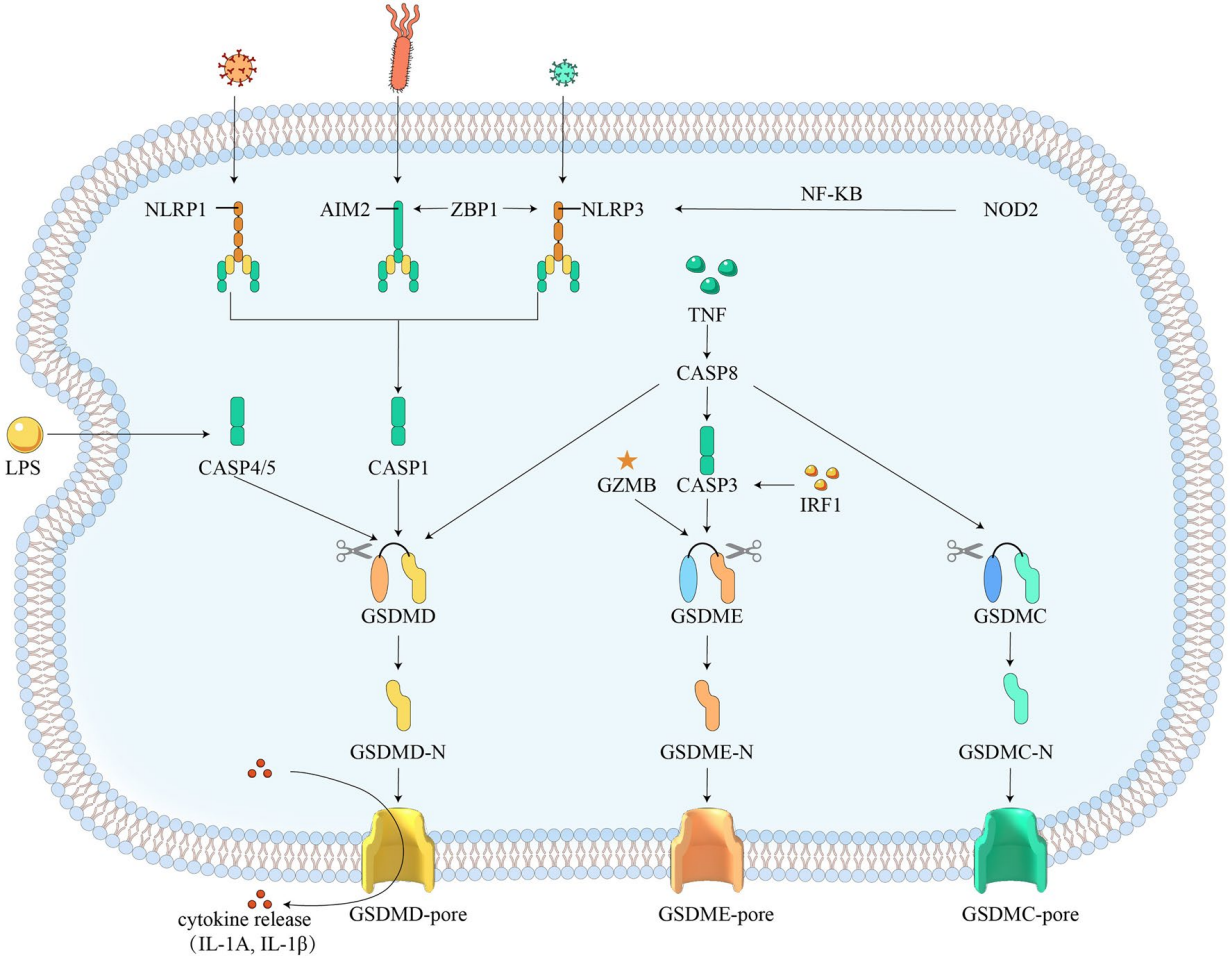
Pyroptosis pathway in IBD.

Firstly, we elucidated the pyroptosis landscape under two disease states. During inflammation, mRNA expression of *AIM2*, *CASP1, CASP4, CASP5, CASP8, GSDMC, GSDMD, GZMB, IL1A, IL1B, IRF1, NLRP1, NLRP3, NOD2, TNF*, and *ZBP1* increases. Based on these genes, we have comprehensively mapped the pyroptosis pathway in inflammatory bowel disease (Fig. 9). Conceivably, GSDMD remains the most important executioner of pyroptosis. Mechanically, once detecting microbial motifs, environmental irritants, or endogenous danger signals, inflammasome sensors (such as NLRP1, NLRP3 and AIM2) recruits recruitment domain (ASC; also called PYCARD) and CASP1 to develop the inflammasome speck, activating CASP1 and processing IL-1β and IL-18, leading to the cleavage of the pore-forming protein GSDMD and the release of inflammatory mediators (IL1A and IL1B)^30^. In addition, CASP4, CASP5, and CASP11 can form the non-canonical inflammasome that cleaves GSDMD and induces pyroptosis when they are activated by lipopolysaccharide (LPS)^31^. ZBP1 can mediate the activation of NLRP3 and participate in inflammasome sensors related panoptosome^32,33^. NOD2, as an intracellular receptor, can activate the NF-κB pathway, which is associated with colonic epithelial pyroptosis^34^. GZMB is a member of granzyme (GZM) family, which can directly cleave GSDME and cause pyroptosis^35^. TNF cleaves GSDME by activating CASP8/CASP3^36^, and IRF1 regulates the expression of CASP3, IRF1 knockout reduces mucosal inflammation induced by TNF^37^. Moreover, CASP8 can cleave GSDMD and GSMDC, and its activity determines the pathway of cell death^38,39^.

Until now, little is known about the cleavage trigger of GSDMC and its role in IBD. Interestingly, we observed an increase in GSDMC mRNA expression in experimental colitis model. We observed increased GSDMC mRNA expression in IBD patients and cleavage of GSDMC was upregulated in experimental colitis mice, suggesting that GSDMC may be involved in IBD, similarly to GSDME and GSDMD. We propose that the role of GSDMC in mediating pyroptosis in IBD is a novel research field worth further exploration.

Further, we ultimately identified and verified five genes (*CAPS1, CASP5, GSDMD, AIM2,* and *NLRP3*) as the hub pyroptosis genes for IBD patients, all of which were upregulated during the inflammatory status and could serve as potential biomarkers for diagnosing the disease. Early identification of non-responders to anti-TNF therapy remains a major unresolved clinical challenge. Therefore, we further investigated the expression changes of these genes in response to anti-TNF therapy. Our results show that AIM2 has the most satisfactory predictive value for distinguishing whether patients respond or not. In addition, most hub genes expressed decreased in responders during the treatment, whereas there was no significant alteration in the treatment of non-responders, inferring anti-TNF agents could reduce the expression of pyroptosis-related genes, yet elevated pyroptosis burden (particularly when AIM2 is highly expressed) may be associated with diminished response to anti-TNF antibodies. We then validated the conclusion in human samples and found that the levels of the AIM2 protein were higher in non-responders than in responders, supporting our bioinformatics results.

AIM2 (absent in melanoma 2) is well known for playing a protective role in many malignancies^40^. However, the role of AIM2 as the sensor of inflammasome in inflammatory diseases has yet to be fully elucidated. Intriguingly, limited reports found that AIM2 activation could protect intestinal epithelium by maintaining homeostasis, and AIM2 knockout exacerbates experimental colitis induced by DSS^41,42^. Instead, recent studies pointed out that AIM2 activation aggravates pyroptosis and causes serious tissue damage in various inflammatory disorders^43–45^. We hypothesize that AIM2 also enhances inflammation by exacerbating intestinal injury in human colitis. Consequently, we conducted an in-depth bioinformatics analysis of the function of AIM2 inflammasome. Based on unsupervised consensus clustering, patients were divided into two subtypes. Cluster 2 with higher expressions of AIM2 inflammasome and proportion of non-responders exhibited abundant immune cell infiltration and activated immune reaction, and multiple immune-related pathways were activated according to functional enrichment analysis. We propose that AIM2 may play the pro-inflammatory role in human colitis while genetic ablation in an experimental colitis model could result in mice being unable to resist microbial invasion, leading to more severe tissue damage.

## Conclusion

In conclusion, we systematically summarized the landscape of pyroptosis in inflammatory bowel disease and identified *AIM2, CASP1, CASP5, GSDMD,* and *NLRP3* as hub genes. We discovered AIM2 as a potential biomarker for predicting the efficacy of anti-TNF therapy. A stratification of IBD patients based on AIM2 inflammasome-associated gene expression revealed differential immunological signature between distinct clusters. Our findings provide new insights into the pathogenesis of IBD and may contribute to clinical decision-making processes.

### Supplementary Information

Supplementary Figure S1.

Supplementary Figure S2.

Supplementary Figure S3.

Supplementary Figure S4.

Supplementary Information.

Supplementary Legends.

Supplementary Table S1.

Supplementary Table S2.

Supplementary Table S3.

## Data Availability

Codes and other data are available from the corresponding author upon reasonable request.

## Abbreviations

UC: Ulcerative colitis
CD: Crohn’s disease
IBD: Inflammatory bowel disease
DEGs: Differentially expressed genes
MCODE: Molecular complex detection
GSDMD: Gasdermin D
CDi: Crohn’s ileitis
CDc: Crohn’s colitis
PPI: Protein–protein interaction
ROC: Receiver operating characteristic curves
AUC: Area under the curve
GO: Gene ontology
KEGG: Kyoto encyclopedia of genes and genomes
GSEA: Gene set enrichment analysis
DSS: Dextran sulphate sodium
PVDF: Polyvinylidene fluoride
IOD: Integral optical density
TNBS: Trinitrobenzene sulfonic
LPS: Lipopolysaccharide
