# Supplementary figures and images for: Pyroptosis burden is associated with anti-TNF treatment outcome in inflammatory bowel disease: new insights from bioinformatics analysis
Source: Sci Rep. 2023 Sep 22;13:15821. doi: 10.1038/s41598-023-43091-0 (PMC10516897; doi:10.1038/s41598-023-43091-0)

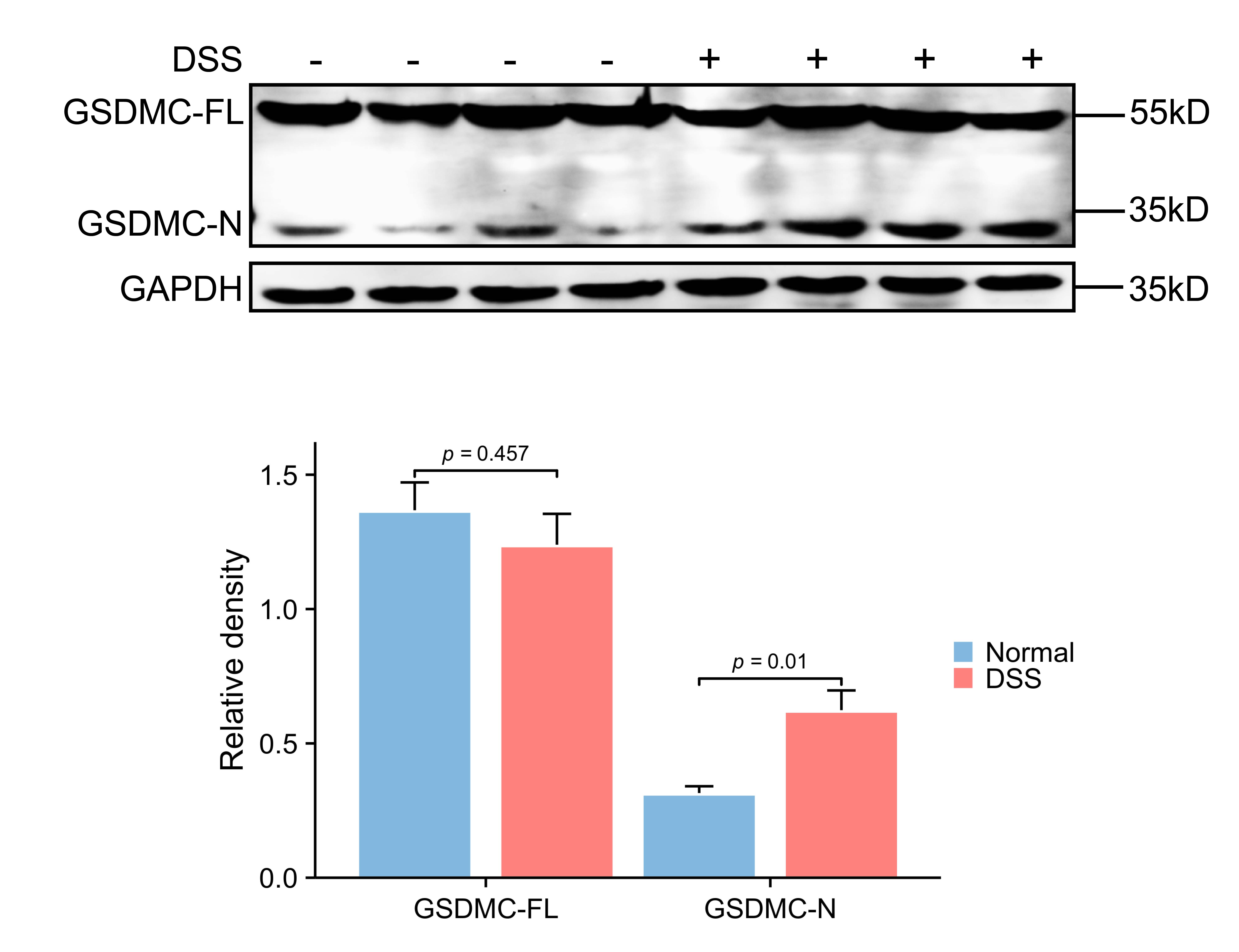

Supplement: Supplementary file 1 — Supplementary Figure S1. [file 41598_2023_43091_MOESM1_ESM.jpg]

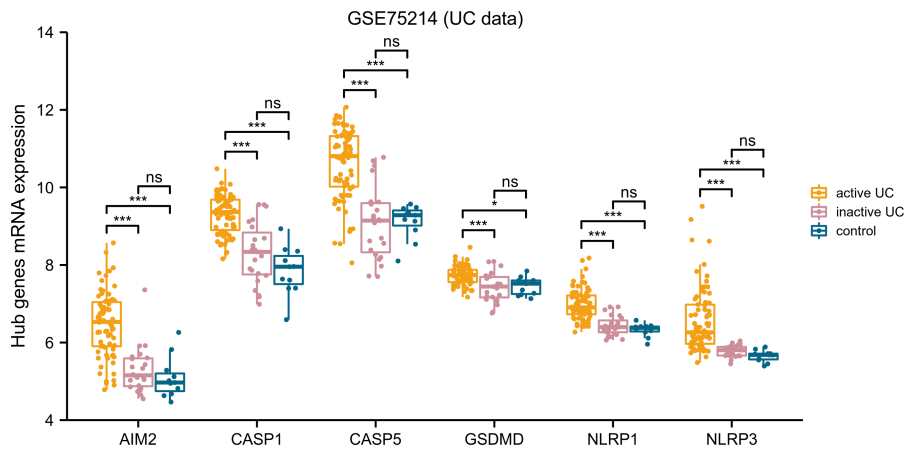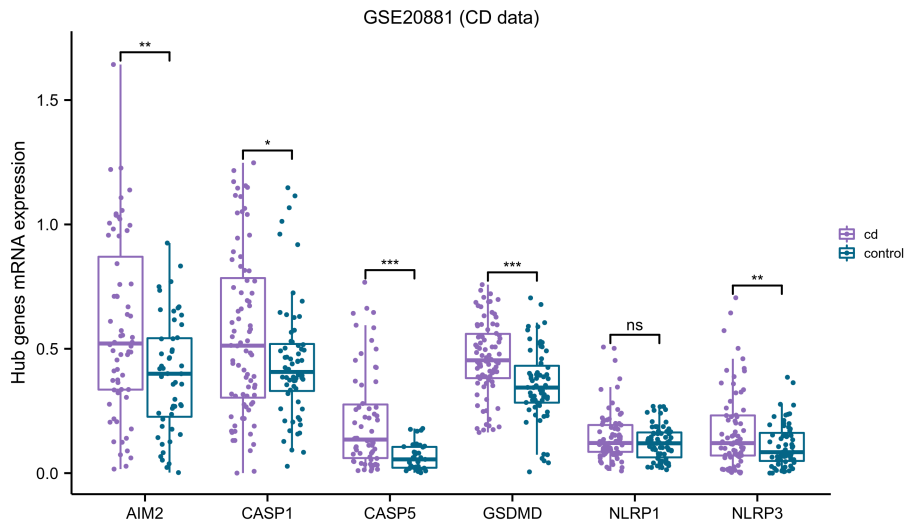

Supplement: Supplementary file 2 — Supplementary Figure S2. [file 41598_2023_43091_MOESM2_ESM.pdf]

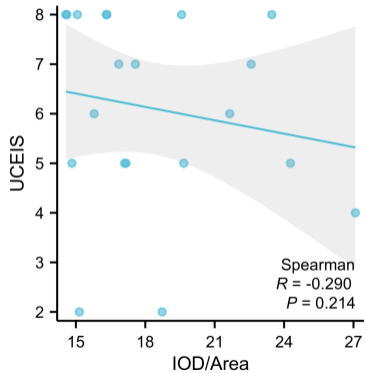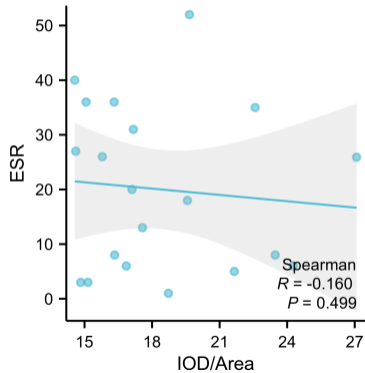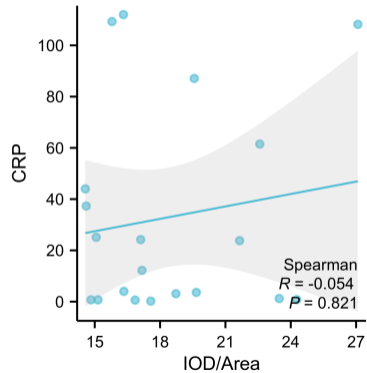

Supplement: Supplementary file 3 — Supplementary Figure S3. [file 41598_2023_43091_MOESM3_ESM.pdf]

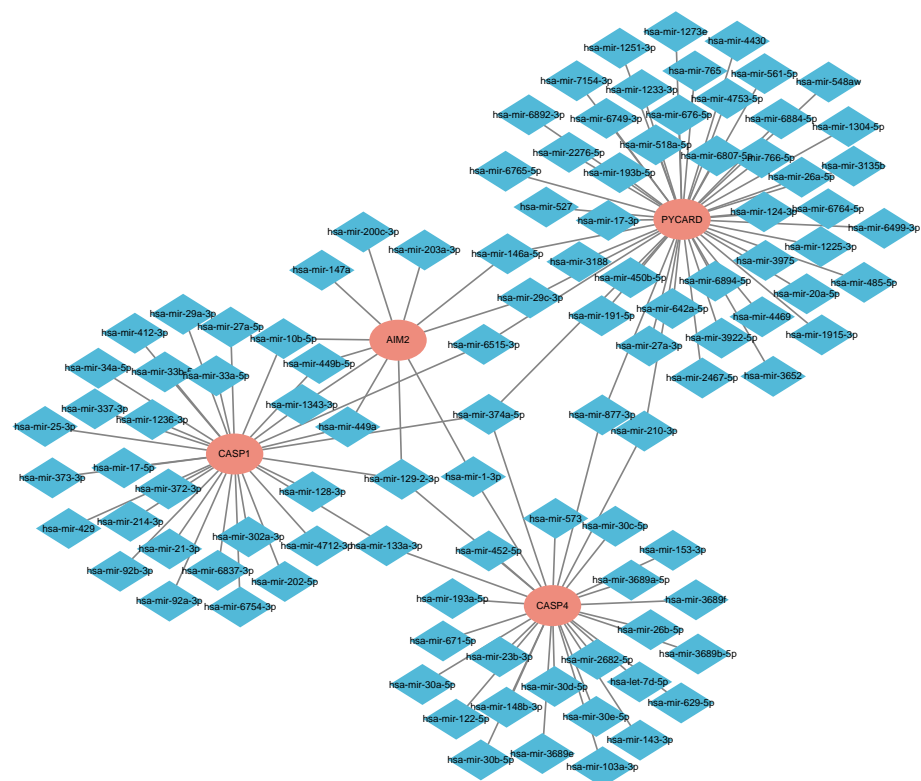

Supplement: Supplementary file 4 — Supplementary Figure S4. [file 41598_2023_43091_MOESM4_ESM.pdf]
