# Supplementary material for: Pyroptosis burden is associated with anti-TNF treatment outcome in inflammatory bowel disease: new insights from bioinformatics analysis
Source: Sci Rep. 2023 Sep 22;13:15821. doi: 10.1038/s41598-023-43091-0 (PMC10516897; doi:10.1038/s41598-023-43091-0)
Supplement: Supplementary file 5 — Supplementary Information. [file 41598_2023_43091_MOESM5_ESM.pdf]

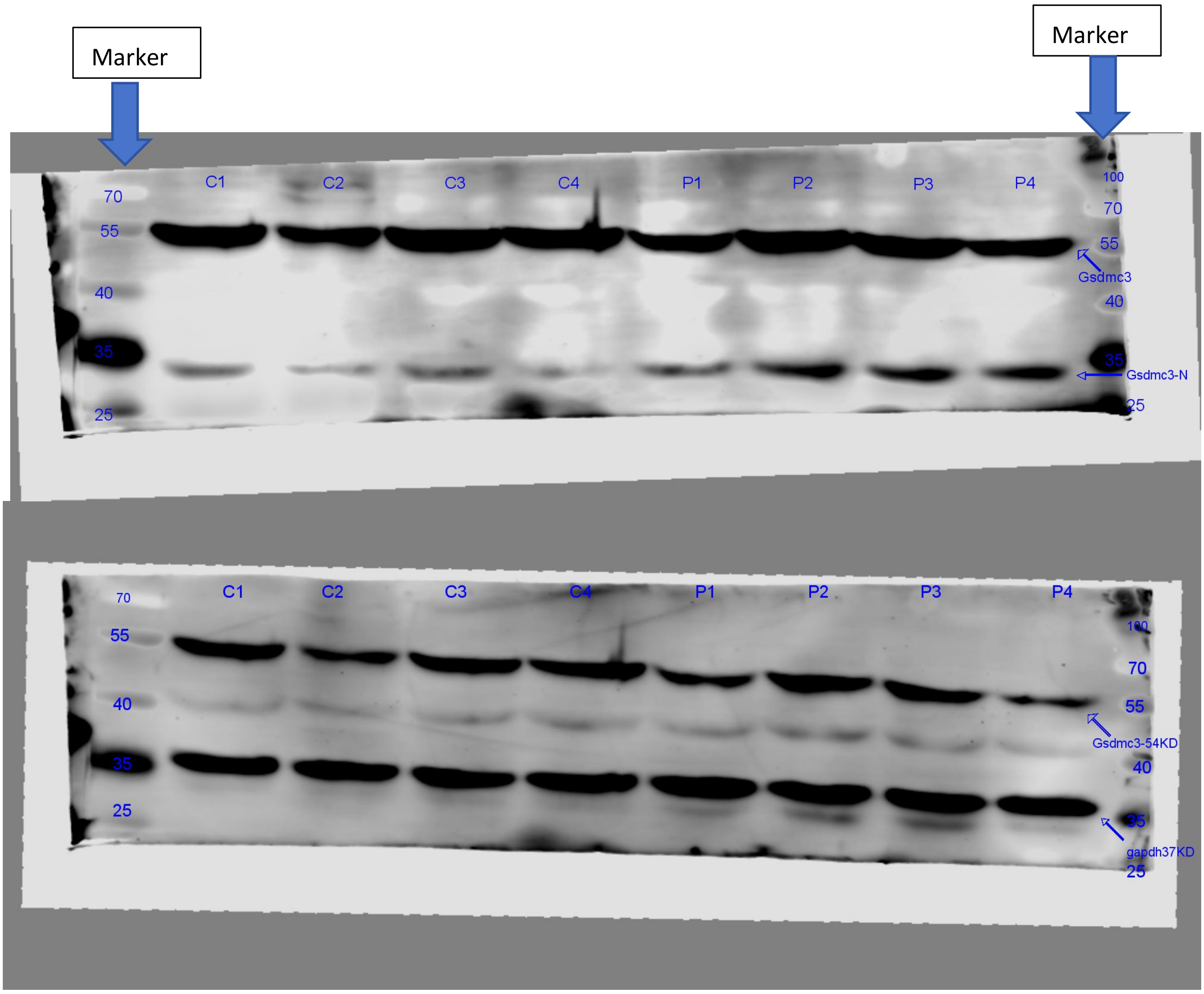

This is a membrane containing GSDMC, GSDMC-N, and GAPDH. C1-C4 is a control group of four mice, while P1-P4 is four mice treated with DSS, to save antibodies, after protein was transferred from SDS-PAGE gel to a PVDF membrane, we would crop the PVDF membrane into thin bands based on target protein molecular weight, then the blot with separated proteins would be incubated with primary antibodies. So, raw data for western blot are separated blot images. Anti-GSDMC3 antibody purchased from Abuclonal, Cat # A16741, the characteristics of antibody and cleavage bands have been described in this study (Zhao M, Ren K, Xiong X, Xin Y, Zou Y, Maynard JC, Kim A, Battist AP, Koneripalli N, Wang Y, et al: Epithelial STAT6 O-GlcNAcylation drives a concerted anti-helminth alarmin response dependent on tuft cell hyperplasia and Gasdermin C. Immunity 2022, 55:623-638.e625.)
