# Supplementary material for: Pyroptosis burden is associated with anti-TNF treatment outcome in inflammatory bowel disease: new insights from bioinformatics analysis
Source: Sci Rep. 2023 Sep 22;13:15821. doi: 10.1038/s41598-023-43091-0 (PMC10516897; doi:10.1038/s41598-023-43091-0)
Supplement: Supplementary file 6 — Supplementary Legends. [file 41598_2023_43091_MOESM6_ESM.docx]

**Figure legends**

**Figure S1.** Elevated expression of GSDMC-N in experimental colitis mice. The blots were cut prior to hybridisation with antibodies during blotting. Original blots are presented in supplementary file. Anti-GSDMC3 antibody purchased from Abuclonal, Cat # A16741）

**Figure S2.** Validation of the expression of hub genes.

**Figure S3.** The relationship between AIM2 protein and clinical indicators.

**Figure S4.** Construction of the miRNA-mRNA network using the miRNet programme and Cytoscape.

**Table legends**

**Table S1.** Pyroptosis genes list.

**Table S2.** Functional enrichment analysis.

**Table S3.** MiRNA-mRNA regulatory network.
